# *OsFH3* Encodes a Type II Formin Required for Rice Morphogenesis

**DOI:** 10.3390/ijms222413250

**Published:** 2021-12-09

**Authors:** Shuwei Chang, Zhanhong Ren, Chang Liu, Pingzhou Du, Jingbin Li, Zengyu Liu, Fengli Zhang, Haili Hou, Jianxin Shi, Wanqi Liang, Litao Yang, Haiyun Ren, Dabing Zhang

**Affiliations:** 1 Joint International Research Laboratory of Metabolic and Developmental Sciences, School of Life Sciences and Biotechnology, Shanghai Jiao Tong University, Shanghai 200240, China; shuweichang@sjtu.edu.cn (S.C.); lijingbin528@gmail.com (J.L.); zengyu.liu@outlook.com (Z.L.); zhangfengli@sjtu.edu.cn (F.Z.); houhaili0372@sjtu.edu.cn (H.H.); jianxin.shi@sjtu.edu.cn (J.S.); wqliang@sjtu.edu.cn (W.L.); yylltt@sjtu.edu.cn (L.Y.); 2 Hubei Key Laboratory of Diabetes and Angiopathy, Medicine Research Institute, Xianning Medical College, Hubei University of Science and Technology, Xianning 437100, China; renzhanhong@hbust.edu.cn; 3 Key Laboratory of Cell Proliferation and Regulation of Ministry of Education, College of Life Science, Beijing Normal University, Beijing 100875, China; chang.liu@bnu.edu.cn (C.L.); dupingzhou@bnu.edu.cn (P.D.); 4 School of Agriculture, Food and Wine, University of Adelaide, Waite Campus, Urrbrae, SA 5064, Australia

**Keywords:** OsFH3, morphological defects, profilin–actin complex, localization, rice

## Abstract

The actin cytoskeleton is crucial for plant morphogenesis, and organization of actin filaments (AF) is dynamically regulated by actin-binding proteins. However, the roles of actin-binding proteins, particularly type II formins, in this process remain poorly understood in plants. Here, we report that a type II formin in rice, *Oryza sativa* formin homolog 3 (OsFH3), acts as a major player to modulate AF dynamics and contributes to rice morphogenesis. *osfh3* mutants were semi-dwarf with reduced size of seeds and unchanged responses to light or gravity compared with mutants of *osfh5*, another type II formin in rice. *osfh3 osfh5* mutants were dwarf with more severe developmental defectiveness. Recombinant OsFH3 could nucleate actin, promote AF bundling, and cap the barbed end of AF to prevent elongation and depolymerization, but in the absence of profilin, OsFH3 could inhibit AF elongation. Different from other reported type II formins, OsFH3 could bind, but not bundle, microtubules directly. Furthermore, its N-terminal phosphatase and tensin homolog domain played a key role in modulating OsFH3 localization at intersections of AF and punctate structures of microtubules, which differed from other reported plant formins. Our results, thus, provide insights into the biological function of type II formins in modulating plant morphology by acting on AF dynamics.

## 1. Introduction

Microfilaments and microtubules are two important cytoskeletal components in plant cells. Microfilaments are actin filaments (AF) composed of polymerized globular actin (G-actin) monomers. The initial nucleation of monomers to form a new AF, and subsequent AF elongation, bundling of filaments into cables, and cross-interaction of cables are all mediated by actin-binding proteins. In vivo, actin monomers bind to the small protein profilin, which inhibits spontaneous nucleation and elongation to sustain homeostasis, and serves as a nucleotide exchange factor for actin, increasing the exchange of ATP or ADP at least fivefold [1,2].

The AF network in plants plays indispensable roles in many biological processes, including morphogenesis [3], signal transduction [4,5], stomatal opening and closing [6], hormone signaling [7,8], immunity [9,10], organelle transport [11,12], cell division [13], and cell growth [14]. Several classes of actin-binding proteins have been implicated in AF formation, including formins, actin-related proteins-2/3 (Arp2/3), capping proteins, and enabled/vasodilator-stimulated phosphoproteins (Ena/VASP) [15,16,17,18,19].

Formins in higher plants are divided into two types according to their N-terminal domains [20,21]. Type I formins contain a transmembrane domain and are associated with cell wall proteins [21]; type II formins contain a phosphatase and tensin homolog (PTEN) domain essential for interaction with membranes [3,20,22].

Both types contain C-terminal FH1 and FH2 domains, which are evolutionarily conserved [23,24]: the FH1 domain promotes AF elongation by binding to profilin–actin monomers via its polyproline domains [25,26], while the FH2 domain is essential for AF nucleation [3,27].

Compared with the known functions of type I formins, for example, in affecting pollen tube growth [27], cytokinesis [28], membrane trafficking pathways [29], and root cell ontogeny [30], the function of type II formins is largely unknown. Single knockouts of formin genes in plants generally exhibit only mild phenotypes, likely due to functional redundancy between formin family proteins [31,32,33,34]. Knockouts of two type II formins have been reported in moss; while single mutants do not show any obvious phenotype, double mutants are severely stunted with disrupted actin organization [35].

The model dicot plant *Arabidopsis thaliana* has 21 predicted formins, including 10 type II members. Among characterized type II formins, AtFH14 is involved in AF-microtubule interactions, regulating their dynamics during cell division [36], while AtFH13 and its distant homolog AtFH14 form heterodimers to associate with microtubules and the periphery of the endoplasmic reticulum [37].

The model monocot rice has five type II formins. Among them, only OsFH5 has been functionally characterized; OsFH5 acts as a core regulator of actin nucleation and AF elongation and associates with the microtubule network [3,38]. Loss-of-function *osfh5* mutants exhibit serious defects in vegetative and reproductive growth [3], such as delayed cell growth [39], stronger sensitivity to gravity in root [40], and abnormal shoot gravitropism [41]. In addition, OsFH15, a rice type I formin, interacts with both AF and microtubules to regulate grain size by affecting cell expansion [33].

In this paper, we describe the biochemical and biological functions of another type II formin, OsFH3, a homolog of OsFH5. We show that functions of OsFH3 and OsFH5 in rice morphogenesis are partially redundant. OsFH3, localized at intersections of the actin cytoskeleton and punctate structures of microtubules, can nucleate actin, bundle AF, and bind but not bundle microtubules. Our results demonstrate that OsFH3 contributes to rice morphology by modulating the AF dynamics.

## 2. Results

### 2.1. Phenotypes of osfh3 Mutant

To understand the function of OsFH3, we used the 2 kb *OsFH3* promoter to drive the expression of the β-glucuronidase (GUS) reporter gene (Figure S1). GUS signals were detected in root, stem, leaf, and pedicle but not in root tips and anthers. The ubiquitous expression profile of *OsFH3* is, thus, consistent with those of most reported rice formins [38]; however, it is discrete from that of reported type II formin gene *OsFH5*, whose expression is observed in root tips but not in pedicle [3].

**Figure 1:**
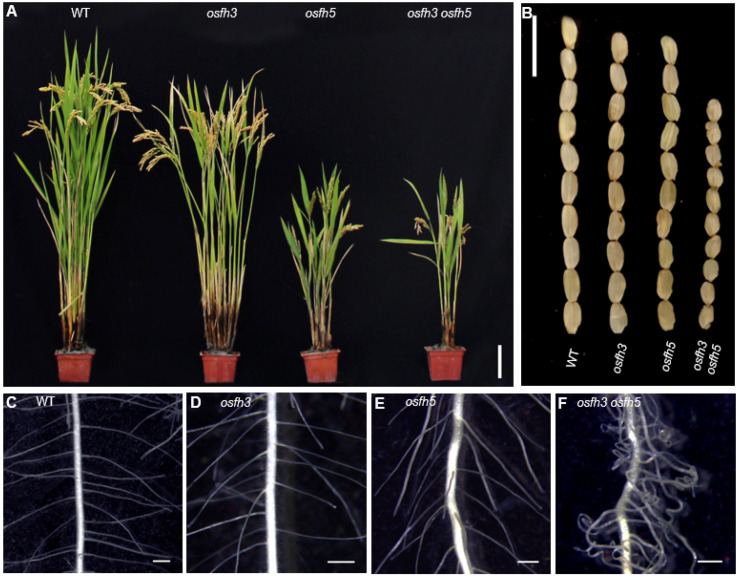
Phenotypes of WT, *osfh3*, and *osfh5* single and double mutant plants. (**A**) Plant height at grain maturation. Bar = 10 cm. (**B**) Mature grains. Bar = 1 cm. (**C**–**F**) Lateral roots of 3-day old seedlings. Bar = 1 mm.

To determine the molecular function of OsFH3, two loss-of-function *osfh3* mutants were generated using CRISPR/Cas9 targeting in the second exon of the N-terminal PTEN domain, which caused premature protein truncation in wild-type (WT) plants (Figure S1A). Because of OsFH3 and OsFH5, the two of the known five type II formins in rice [42], four CRISPR/Cas9-induced *osfh3* mutants (targeting the same sequence) were generated in an available *osfh5* line (also called *rice morphology determinant1-1*, *rmd1-1* [3]). These resulting *osfh3osfh5* double mutants were then used to investigate their functional redundancy. The *osfh3* mutant was semi-dwarf, while the *osfh5* and *osfh3 osfh5* mutants exhibited more severe phenotypes: dwarf with smaller seeds and curved roots (Figure 1A; Figure S2A,B).

Other phenotypes, such as seed length, panicle primary branch numbers, and root hair curling, were affected by *OsFH3* and *OsFH5* independently. Compared with WT plant, mutants exhibited shorter seed length and reduced primary branches in the inflorescence: seed length of the single and double mutant was about 94% and 73% of that of WT plant, respectively (Figure 1B and Figure S2C); and the number of primary branches in the inflorescence of the single and double mutant was about 80% and 58% of that of WT plant, respectively (Figure S2D,E). Compared with WT and *osfh3* or *osfh5* single mutants, lateral roots of *osfh3 osfh5* double mutants were much curlier (Figure 1C–F). These results indicate that both *OsFH3* and *OsFH5* play important roles in regulating rice morphogenesis and that knockout of *OsFH3* exacerbates the phenotypic defects in the *osfh5* mutant background.

**Figure 2:**
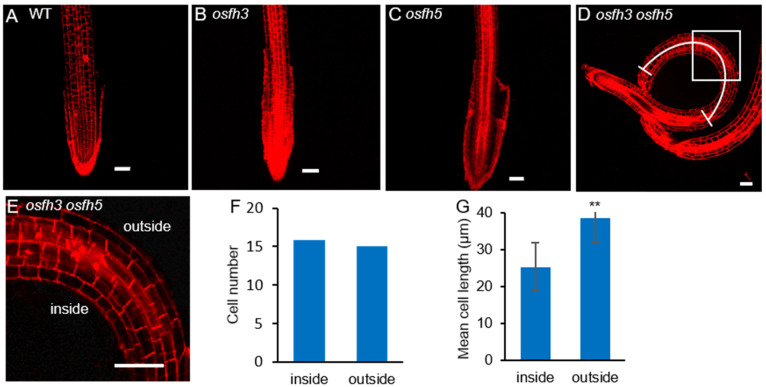
Propidium iodide staining results of WT, *osfh3*, *osfh5*, and *osfh3 osfh5* roots tips. (**A**–**D**) WT, *osfh3*, *osfh5*, and *osfh3 osfh5* roots tips were stained with propidium iodide. Bar = 50 µm. (**E**) Enlarged pictures of boxes in the D pictures. (**F**) Cell number statistic of curved segment in *osfh3 osfh5* roots. *n* = 10. (**G**) Cell length statistic of curved segment in *osfh3 osfh5* roots. The arc in D marks the data source area in the root. *n* = 100. ** *p* < 0.01, Student’s *t*-test.

To test the cellular state of roots in mutants, propidium iodide (PI) was used (Figure 2A–E). The staining results showed that changes in cell numbers between the inside and the outside walls of curved roots were not significant (Figure 2F), while the cell length located inside of the curved roots became shorter than that located outside of the curved roots in *osfh3 osfh5* mutant lines (Figure 2D,G). However, such a cell length difference between the inside and the outside wall of roots was not observed in other plants, including WT, *osfh3*, and *osfh5*. These results indicated that root curvature observed in *osfh3 osfh5* is caused by changes in the length but not the number of cells on the opposite side of the root.

Previous studies reported that *osfh5* mutants are more sensitive to gravity [40], and this response is affected by sunlight [41]. Our results revealed that the sensitivity of *osfh3 osfh5* roots to gravity is similar to that of *osfh5* roots (Figure S3A,B) and that *osfh3* roots do not show an obvious gravitropic phenotype. In addition, double mutant exhibited a different phenotype to light from either single mutant. *osfh3* seedlings (5-d old) grew upright under both light and dark conditions, *osfh5* seedlings (5-d old) grew curved under light but straight under dark conditions, while *osfh3 osfh5* grew bent under both light and dark conditions (Figure S3C,D). Thus, OsFH3 appears to function in gravity-sensing under dark, which is distinct from OsFH5.

### 2.2. OsFH3 Nucleates Actin

**Figure 3:**
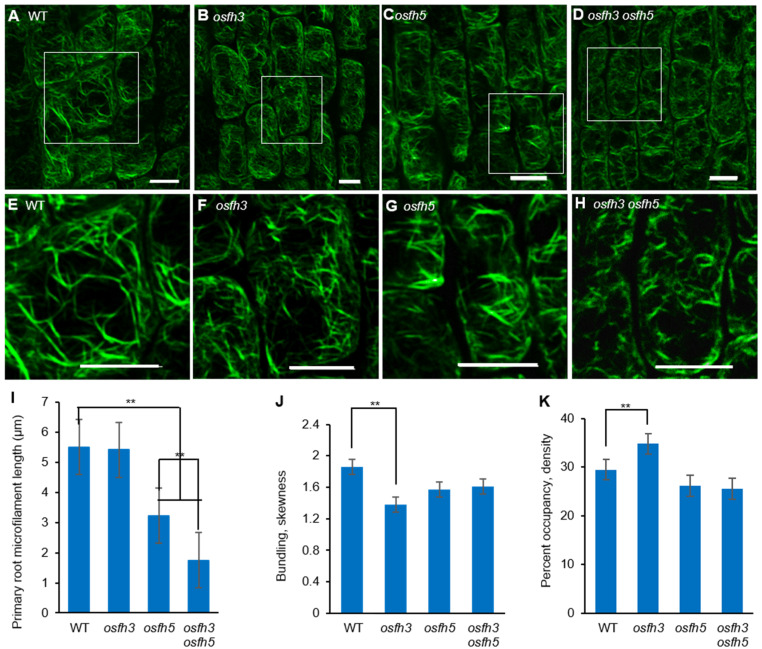
Microfilaments staining results with Alexa Fluor 488^®^-phalloidin. (**A**–**D**) Alexa Fluor 488^®^-phalloidin stained lateral root elongation zone of 3-day-old rice seedlings. Bar = 10 µm. (**E**–**H**) Enlarged pictures of white boxes in A to D, respectively. Bar = 10 µm. (**I**) Length of microfilaments in primary roots. Mean ± SD, *n* > 100 microfilaments from 10 cells across 5 plants. ** *p* < 0.001, one-way ANOVA. (**J**) Bundling (skewness) of 3-day-old seedling roots. Mean ± SD, *n* = 25 cells across 5 plants. ** *p* < 0.001, one-way ANOVA. (**K**) Percentage of occupancy (Actin filament abundance) of 3-day-old seedling roots. Mean ± SD, *n* = 25 cells across 5 plants. ** *p* < 0.001, one-way ANOVA. Region selection was divided by cells of pictures from elongation zone and quantified with ImageJ.

To obtain the mechanistic insights into the function of OsFH3 in rice morphology, Alexa Fluor 488^®^-phalloidin was used to stain AF in the lateral roots of 3-day-old seedlings (Figure 3A–D) to examine the effect of OsFH3 on actin nucleation and AF connection. While AF length was obviously reduced in *osfh5* and even further reduced in *osfh3 osfh5* lines, there was no obvious reduction in AF length of *osfh3* compared with WT (Figure 3I). In addition, while decreased bundling value and increased AF abundance were observed in *osfh3* (Figure 3J,K), such changes were not found in *osfh5* or double mutant.

**Figure 4:**
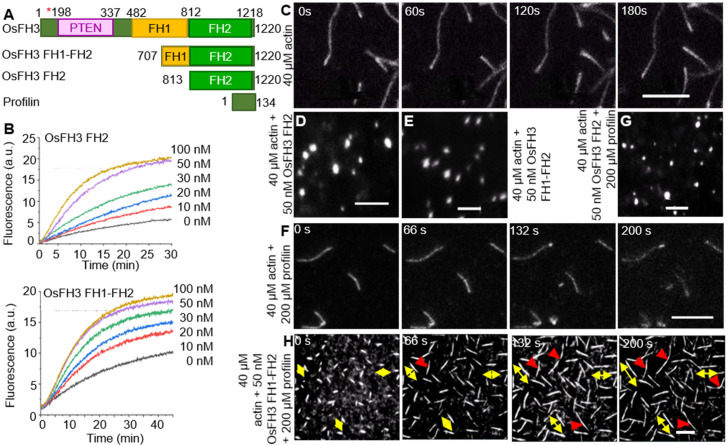
OsFH3 nucleates actin and promotes formation and connection of AF. (**A**) Schematic diagram of OsFH3 structure, indicating FH1–FH2 and FH2 constructs used for actin testing. Amino acid positions in the full-length protein are given. Red asterisk indicates mutation position in *osfh3* mutants. PTEN, phosphatase, and tensin homolog domain. Profilin is PRF4 from Arabidopsis. (**B**) Time course of actin nucleation in the presence of OsFH3 FH2 (above) or OsFH3 FH1–FH2 (below). Pyrene fluorescence (a.u., arbitrary units) is proportional to nucleation. (**C**–**E**) The effect of OsFH3 domains on AF elongation visualized by time-lapse fluorescence microscopy. Fluorescence shows Oregon Green-labeled actin, showing AF elongation, in the actin-only experiment (**C**); and stalled elongation of AF (**D**,**E**) in the presence of OsFH3 FH2 and OsFH3 FH1–FH2 protein segments. Bar = 5 µm. (**F**–**H**) The effect of OsFH3 domains on AF elongation and bundling in the presence of profilin visualized by time-lapse fluorescence microscopy. Fluorescence shows Oregon Green-labeled actin. Actin and profilin only (**F**); actin, profilin, and OsFH3 FH2 (**G**); actin, profilin, and OsFH3 FH1–FH2 (**H**). Yellow arrows in H indicate bi-directional elongation of AF. Red arrows indicate connection of AF. Bar = 5 µm.

To further unravel the biological role of OsFH3, expressing the full length of OsFH3 in *E. coli* was performed to explore OsFH3′s function in actin assembly, which, unfortunately, failed to generate functional protein, likely due to the difficulty in expressing 15 highly repetitive polyproline segments within the FH1 domain. As an alternative approach, the truncated FH1–FH2 segment (containing four polyproline segments) and the FH2 domain were expressed and purified (Figure 4A). These protein segments were incubated in the presence of actin monomers to examine their effects on actin assembly. Both OsFH3 FH2 and OsFH3 FH1–FH2 decreased the initial lag of actin monomer association in a dose-dependent manner, indicating their active roles in actin nucleation (Figure 4A,B).

Time-lapse fluorescence microscopy was used to directly visualize AF elongation in the presence of OsFH3 protein segments. AF nucleation and elongation proceeded normally in the control experiment without the addition of OsFH3 proteins (Figure 4C and Supplemental Movie S1). However, when OsFH3 FH2 or OsFH3 FH1–FH2 was added, small bright spots were observed, indicating that actin nucleation could occur, but elongation could not (Figure 4D,E; Supplemental Movies S2 and S3). When profilin was added to the initial actin monomers, OsFH3 FH1–FH2 could promote AF elongation, but OsFH3 FH2 domain could not, likely due to its lack of polyproline sequences (Figure 4F–H). In the presence of profilin and OsFH3 FH1–FH2, the AF appeared to grow from both ends (Figure 4H, bi-directional yellow arrows; Supplemental Movie S4), compared with only one direction of growth in the control experiment (Figure 4F). Moreover, when different AF extended to connecting points, the connecting ends of the AF ceased to elongate (Figure 4H, red arrows; Supplemental Movie S4). These results indicate that OsFH3 protein is required for normal AF elongation and organization in the presence of the profilin–actin complex.

### 2.3. OsFH3 Bundles and Caps AF

**Figure 5:**
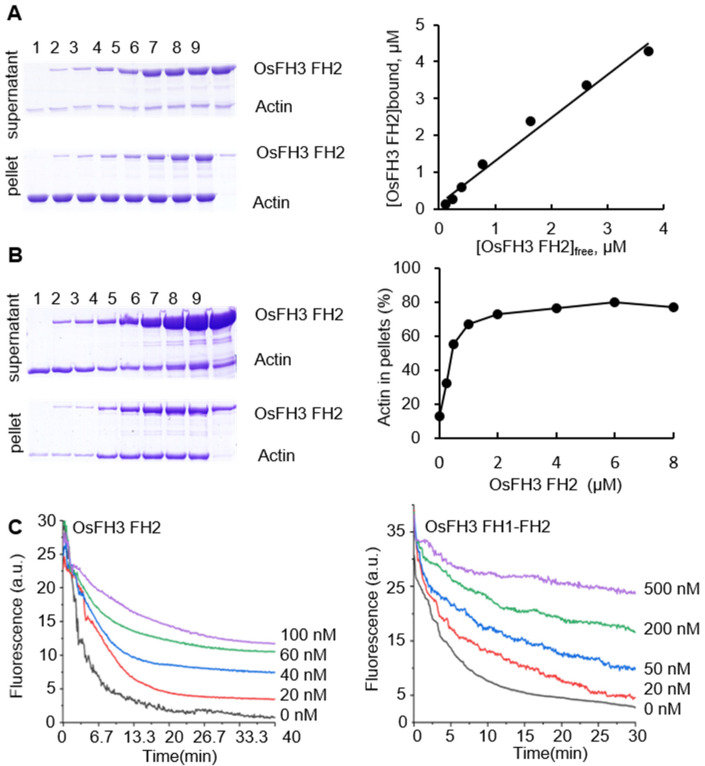
OsFH3 FH2 domain bundles and caps AF. (**A**) Determination of AF binding to OsFH3 FH2 using high-speed co-sedimentation assays. Lanes 1–8, 5 µM actin with 0, 0.25, 0.5, 1, 2, 4, 6, and 8 µM OsFH3 FH2, respectively; lane 9, 8 µM OsFH3 FH2, no actin. Graph (**left**) shows quantification of Coomassie staining on protein gel (**right**). (**B**) Determination of AF binding to OsFH3 FH2 using low-speed co-sedimentation assays. Lanes 1–8, 5 µM actin with 0, 0.25, 0.5, 1, 2, 4, 6, and 8 µM OsFH3 FH2, respectively; lane 9, 8 µM OsFH3 FH2, no actin. Graph (**left**) shows quantification of Coomassie staining on protein gel (**right**). (**C**) Kinetics of AF depolymerization in the presence of OsFH3 FH2 (**left**) or OsFH3 FH1–FH2 (**right**). Pyrene fluorescence (a.u., arbitrary units) is proportional to actin polymer concentration.

To explore the effect of OsFH3 on AF binding and bundling, high- and low-speed co-sedimentation in vitro assays was performed using OsFH3 FH2 and OsFH3 FH1–FH2 proteins. In the high-speed assay, OsFH3 FH2 accumulated in the pellets in proportion to its initial concentration in solution, but only in the presence of actin (Figure 5A), indicating that OsFH3 FH2 can directly bind AF. In low-speed co-sedimentation, the amounts of AF in the pellet reached a maximum level with 2 μM OsFH3 FH2 protein (Figure 5B), demonstrating the bundling ability of OsFH3 FH2. The same results for both high- and low-speed assays were also observed for the OsFH3 FH1–FH2 protein (Figure S4), indicating that both OsFH3 FH2 and OsFH3 FH1–FH2 can bind and bundle AF.

In view of other reports of formins’ capping ability [3,43], dilution-mediated AF depolymerization assays were performed. OsFH3 FH2 and OsFH3 FH1–FH2 segments were both observed to retard actin depolymerization in a dose-dependent manner (Figure 5C), suggesting that OsFH3 might cap and protect the barbed end of AF.

### 2.4. PTEN Domain Affects the Localization of OsFH3

**Figure 6:**
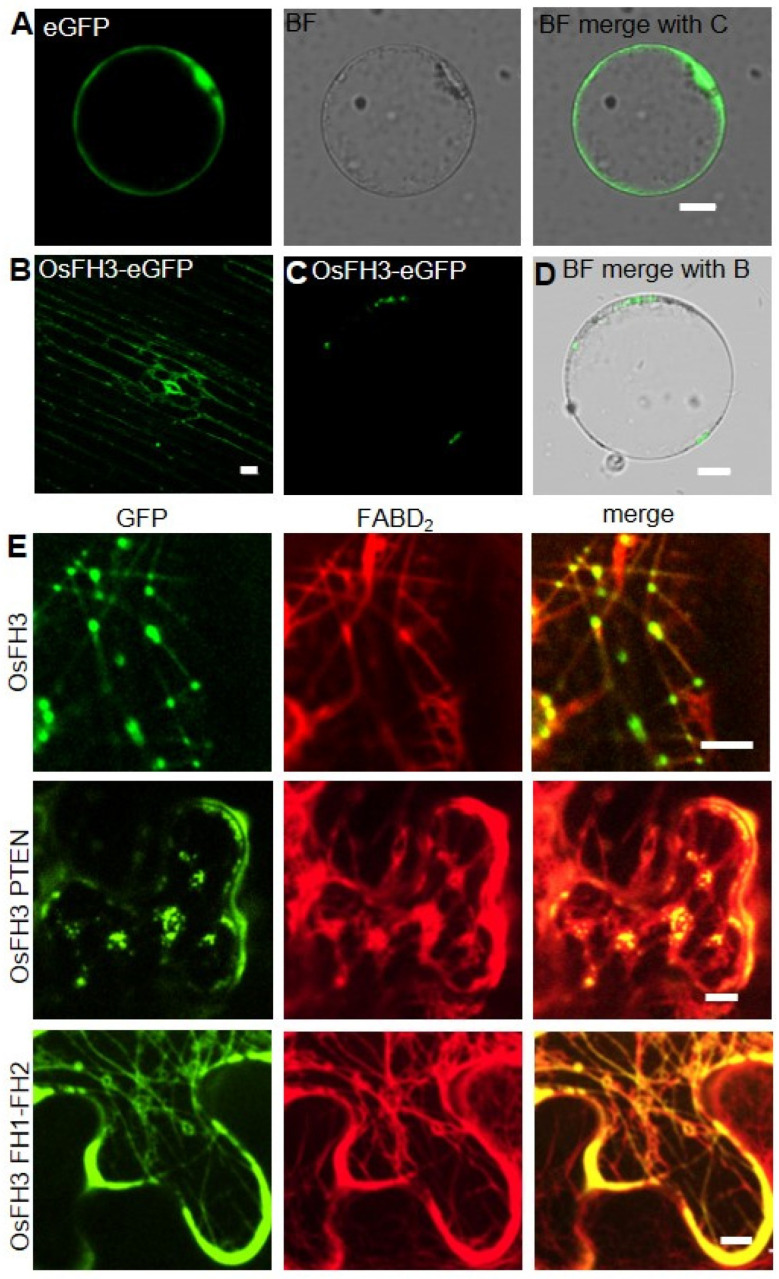
Colocalization of OsFH3 with AF in vivo. (**A**) Contrast, eGFP signals in rice protoplast. Bar= 10 µm. (**B**) OsFH3-eGFP signals in rice coleoptile cells. Bar = 10 µm. (**C**,**D**) OsFH3-eGFP signals in rice protoplast showing GFP (**B**) and merged bright field (BF) with GFP (**C**) images. Bar = 10 µm. (**E**) Colocalization of OsFH3-eGFP full length protein, OsFH3 PTEN-eGFP and OsFH3 FH1-FH2-eGFP domains with AF marker mScarlet-FABD_2_ in tobacco leaves. Bar = 5 µm.

An OsFH3-eGFP fusion protein was used to examine the subcellular localization of OsFH3 in transgenic rice lines and protoplasts. Punctate fluorescent signals were detected in the cytoplasm of rice coleoptile cells and protoplasts (Figure 6B,C). In order to figure out what these dot signals are and given the interaction between OsFH3 and AF, *OsFH3-eGFP* and the AF marker *mScarlet-FABD_2_* were co-expressed in tobacco leaves, for the reason that co-expression of OsFH3-eGFP and AF marker experiment was failed and rice protoplasts could not clearly display the morphology of microfilaments and microtubules (rice protoplasts lost cell wall binding and vacuoles expand to squeeze the cytoplasm into a very small space). These results revealed that most OsFH3 colocalized with AF, predominantly at the intersections of the AF cytoskeleton (Figure 6D and Supplemental Movie S5).

Considering that the PTEN domains of type II plant formins guide protein localization and allow FH1-FH2 binding to AF [3], the OsFH3 protein was split into two halves, the N-terminal PTEN domain (aa 1 to 337) and the C-terminal FH1–FH2 domain (aa 551 to 1218), and each was fused to eGFP. When co-expressed with mScarlet-FABD_2_ in tobacco leaves, PTEN-eGFP co-localized with AF as the full-length protein did, but the AF became disordered (Figure 6E). The FH1–FH2-eGFP protein was also found to be localized across the whole length of highly ordered AF (Figure 6E). These results suggest that the PTEN domain plays a key role in OsFH3 accumulation at intersections of the AF network, while the FH1–FH2 domain helps to bind AF. Thus, PTEN and FH1–FH2 domains work together to determine the precise location of OsFH3 in vivo.

### 2.5. OsFH3 Binds Microtubules

**Figure 7:**
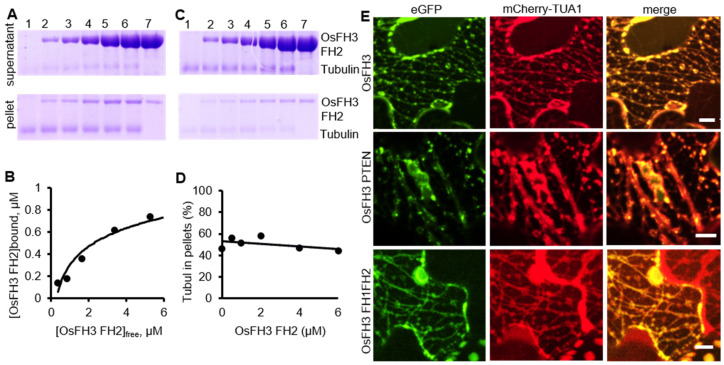
OsFH3 binds and colocalizes with microtubules. (**A**) High-speed co-sedimentation assays to determine tubulin binding to OsFH3 FH2. Lanes 1–6, 2 µM microtubule with 0.5, 1, 2, 4, and 6 µM OsFH3 FH2, respectively; lane 7, 6 µM OsFH3 FH2, no tubulin. (**B**) Graph shows quantification of Coomassie staining on protein gel (**A**). (**C**) Low-speed co-sedimentation assays to determine tubulin bundling with OsFH3 FH2. Lanes 1–6, 2 µM tubulin with 0.5, 1, 2, 4, and 6 µM OsFH3 FH2, respectively; lane 7, 6 µM OsFH3 FH2, no tubulin. (**D**) Graph shows quantification of Coomassie staining on protein gel (**C**). (**E**) Colocalization of OsFH3-eGFP full-length protein, PTEN, and FH1–FH2 domains with microtubule marker mCherry-TUA1 in tobacco leaves. Bar = 5 µm.

Previous reports revealed that some formins, such as AtFH4, AtFH14 in Arabidopsis [37,44], and OsFH5 in rice [3], can bind and bundle microtubules. As OsFH3 is the homolog of OsFH5, the function for OsFH3 to bind and bundle microtubules was tested using high- and low-speed co-sedimentation in vitro assays with OsFH3 FH2 and OsFH3 FH1–FH2 domains. In the high-speed assays, slight increases of OsFH3 FH2 accumulation in pellets in the presence of microtubule were observed as the concentration of OsFH3 FH2 increased (Figure 7A,B), indicating that OsFH3 FH2 binding ability of microtubules is weaker than AF (Figure 5A,B). In low-speed assays, the content of microtubule and OsFH3 FH2 in the pellet was not positively related to the concentration of OsFH3 FH2 (Figure 7C,D), suggesting that OsFH3 cannot bundle microtubule. The same results for both high- and low-speed assays were also observed for the OsFH3 FH1–FH2 protein (Figure S5), indicating that both OsFH3 FH2 and OsFH3 FH1–FH2 can bind but not bundle microtubules.

Next, OsFH3-eGFP, OsFH3 PTEN-eGFP, or OsFH3 FH1–FH2-eGFP was co-transfected with the microtubule marker mCherry-TUA1 into tobacco leaves to examine the in vivo colocalization. Full-length OsFH3 could colocalize with microtubule in a punctate distribution pattern (Figure 6E and Figure 7E), similar but not identical to the colocalization of OsFH3 with AF. PTEN-eGFP also co-localized with microtubules (Figure 7E), while FH1FH2-eGFP colocalize with mCherry-TUA1 along the full lengths of the microtubules (Figure 7E). These results indicate that OsFH3 might anchor the AF cytoskeleton to microtubules, and again, that the two domains play discrete roles in microtubule binding and organization.

To understand the function of OsFH3 in changing the patterning of microtubules in rice, the microtubule staining assay was conducted. It, however, did not reveal any obvious changes of microtubule between *osfh3* and WT. Notably, the *osfh3 osfh5* double mutant showed similar defects of microtubule to the *osfh5* single mutant (Figure S6), suggesting that OsFH3 does not have a key role in modulating microtubule dynamics, which differs from that of OsFH5.

## 3. Discussion

### 3.1. OsFH3 and OsFH5 Synergistically Regulate Rice Morphogenesis

The cytoskeleton, including AF and microtubules, plays vital roles in cell function and reproduction in animals and plants and relies on essential cytoskeleton-binding proteins [8,45]. Formins are one of the most important kinds of cytoskeleton-binding proteins. Currently, two types of formins have been identified in rice [23], and type II formins contain only five members; among them, only *OsFH5* has been functionally characterized; it regulates actin nucleation and AF elongation, associating with the microtubule network [38,39,40,41,44]. This study focused on another type II formin, *OsFH3.* It is almost ubiquitously expressed throughout rice development, with specific expression differences in root tips and pedicle compared with *OsFH5* (Figure S1) [3]. Therefore, we are curious about if both formins have different functions and if they are functionally redundant. For this aim, we generated both *osfh3* single mutant and *osfh3 osfh5* double mutant using CRISPR/Cas9 targeting the same sequence of *OsFH3* in WT and *osfh5* mutant, respectively.

*osfh3* mutants were semi-dwarf with unchanged responses to light or gravity, while *osfh5* mutants were near dwarf with changed response to light or gravity (Figure 1 and Figure S3) [3,38]. *osfh3 osfh5* double mutants were dwarf with curved roots and much smaller seeds than either single mutant (Figure 1 and Figure S2). Intriguingly, 5-day-old seedlings of double mutants grew bent under both dark and light conditions, which differs from either of the single mutants (Figure S3). These results indicate that while functional redundancy may exist between OsFH3 and OsFH5, OsFH3 plays a discrete role in the regulation of rice morphogenesis.

### 3.2. OsFH3 Acts on AF Organization in Rice

Previous research on formins revealed that most plant formins share basic functions, including actin nucleation and AF elongation and bundling, although differences in functions and affinities do exist. In Arabidopsis, AtFH2 cannot nucleate actin but caps and stabilizes AF [43], while AtFH16 could not promote actin nucleation but can bind and bundle AF in vitro [46]. In rice, in vitro biochemical experiments have shown that OsFH5 FH2 and OsFH5 FH1–FH2 domains can direct AF nucleation and elongation in the absence of profilin [3], while the FH1–FH2 domain can nucleate and elongate AF with profilin–actin complexes [38]. Since both OsHF3 and OsFH5 are type II formins, we assumed that OsFH3 act on AF organization in rice.

Indeed, biochemical data indicate that OsFH3 might have a function in mediating the inter-connection of AF (Figure 4H). OsFH3 FH2 and OsFH3 FH1–FH2 domains can nucleate actin in the absence of profilin but cannot direct AF elongation (Figure 4). When profilin was added, only OsFH3 FH1–FH2 could promote AF elongation with profilin–actin complexes. Interestingly, such AF extension is bidirectional, resulting likely from the linking of microfilament fragments rather than the addition of a single G-actin that drives the elongation of AF. Importantly, under these conditions, connected AF ends are no longer elongated, unlike in the presence of OsFH15, where connection ends continue to elongate [33]. Both domains could also cap actin filaments to retard depolymerization (Figure 5C).

In addition, Alexa Fluor 488^®^-phalloidin staining results of roots (Figure 3G) revealed that OsFH3 plays an essential role in directing AF structure and organization. Although AF lengths in the WT and *osfh3* mutant were similar, AF length in the *osfh3 osfh5* double mutant was significantly different from *osfh5*. Disorder in AF structure and organization caused more severe phenotypes in roots and other tissues of *osfh3 osfh5* double mutants (Figure 3).

### 3.3. OsFH3 PTEN-Domain Directs Protein Locating to Actin Cytoskeleton Intersections

PTEN domains of plant type II formins play important roles in linking the AF and microtubule cytoskeleton with plasma or organelle membranes [21,47,48,49]. AFH14 localizes to the preprophase band, spindle, and phragmoplast during cell division [13]; AtFH13 and AtFH14 associate with the endoplasmic reticulum [37], while OsFH5 associates with the chloroplast [3], all via their PTEN domains. We truncated the OsFH3 protein into different domains and found that the PTEN domain plays a key role in directing the subcellular localization of OsFH3 to intersections of the actin cytoskeleton (Figure 6). PTEN domains of animal formins contain phosphorylatable threonine residues that modulate their ability to alter cell migration [50]. Such a key phosphorylation motif is not generally conserved in plant formins [23] with the exception of OsFH3; OsFH3 contains phosphorylatable serine residues, which may explain the differences in OsFH3 subcellular localization compared with other plant formins. Nevertheless, the real effect of the phosphorylation of OsFH3 on its localization remains to be resolved.

### 3.4. OsFH3 Binds to But Does Not Bundle Microtubules

Many formins bind not only AF but also microtubules, for example, AtFH1 [51], AtFH14 [36], and AtFH16 [46] in Arabidopsis; and OsFH5 and OsFH15 in rice [3,38]. In addition, some formins can also bundle microtubules, such as OsFH5 [3]. Here, eGFP signals detected in the cytoplasm and membrane of rice protoplasts (Figure S7A) could not associate its localization with bundling microtubules. However, co-expressing of eGFP-OsFH5 with AF marker mScarlet-FABD_2_ and microtubule marker mcherry-TUA1 in tobacco leaves, respectively, confirmed the interaction between OsFH5 and microfilaments and microtubules (Figure S7) and showed that the binding ability of OsFH5 to microtubules is more strongly than that of OsFH3. Low-speed assays also confirmed that OsFH3 could not bundle microtubules (Figure 7 and Figure S5), which is different from OsFH5. This assumption was further verified by the results of an in vivo col-localization assay using OsFH3 and microtubule marker TUA1, which showed that OsFH3 locates to the punctate structures of microtubules, different from that of OsFH3 to the intersections of the actin cytoskeleton (Figure 7E compared with Figure 6D). Likely, OsFH3 acts as a scaffold protein to anchor the AF skeleton to microtubules, but more detailed studies are required to confirm this hypothesis.

Based on the abovementioned results, OsFH3 and OsFH5 both play important roles in rice morphogenesis but with different effects on AF dynamics. Both OsFH3 and OsFH5 are capable of binding, bundling, and capping to AF, but OsFH3 has no effects on the extension of AF. In addition, although OsFH5 can bind and bundle microtubules, OsFH3 can only bind microtubules. These differential effects of OsFH3 and OsFH5 on AF dynamics could result from their different expression pattern (Figure S1) and protein localization (Figure 6 and Figure 7).

In summary, this work has identified a type II formin, OsFH3, in rice. It is required for rice morphogenesis via modulating AF dynamics, which exhibits a partially synergistic but also a discrete function from OsFH5, a known formin that is closely associated with the microtubule network during rice development. OsFH3 also shows unique biochemical activities in nucleating actin, promoting AF bundling, maintaining AF dynamics, and binding microtubules compared with other reported formins. In addition, OsFH3 may modulate cytoskeleton organization by affecting the intersections of AF. This work provides new insights into the biological function of type II formins in plant development.

## 4. Materials and Methods

### 4.1. Plant Materials and Growth Conditions

Wild-type rice (*Oryza sativa* cv. 9522) and mutant plants were grown in the paddy fields of Shanghai Jiao Tong University (30° N 121° E) from June to September (the natural growing season) according to standard local practice. Stem lengths were measured, and grains were harvested at maturity. The *osfh5* mutant in the 9522 background was available from previous work [3]. The *osfh3* mutant was generated in 9522 and *osfh5* via CRISPR-Cas9 as previously described [52], using guide RNAs as shown in Supplemental Table S1.

For gravitropic experiments, seeds were placed into germination pouches (brand, Phytotc; size, 17.5 × 12.5 cm), embryo side down, and kept upright underwater at 28 °C for 3d. For root tip angle experiments, pouches were laid horizontally, and roots were imaged with Olympus e-410 digital camera every hour for 4 h. Root angle was determined with software ImageJ. For light/dark growth experiments, pouches were moved to either a 16 h photoperiod or no light incubators at 28 °C, 70% humidity, and plants were grown for 5 d.

### 4.2. AF Staining of Roots

AF were stained using the glycerol method [53]. Briefly, roots from 3 d seedlings were incubated in PEM buffer (100 mM PIPES, 10 mM EGTA, 5 mM MgSO_4_, 0.3 M mannitol, pH 6.9) with 1% (*w*/*v*) glycerol and 6.6 mM Alexa Fluor 488^®^-phalloidin (Invitrogen, Carlsbad, CA, USA) for 30 min. Root tips were observed using a Leica TCS SP5 confocal laser scanning microscope equipped with a 363 1.46–numerical aperture HC PLANs objective. Four independent lines for each genotype were observed, and at least 10 images were recorded for each. Filament lengths were measured using Leica TCS SP5 software.

### 4.3. OsFH3 In Vivo Expression

2-kb OsFH3 promoter before ATG of the first exon with primers FH3-GUSS and FH3-GUSA (Supplemental Table S1), constructed into pCAMBIA 1301 vector, then the plasmid was transfected into *Agrobacterium tumefaciens* EHA 105 after sequencing, the EHA 105 agrobacterium transfected into rice 9522 specie [54], and the identified seedlings were planted in the field.

Plant tissues—whole 7 d seedling, roots from 7 d seedling, and whole flowers at filling stage—were collected, rinsed with 80% (*v*/*v*) acetone, and infiltrated with GUS staining solution (100 mM Na_2_HPO_4_, 50 mM KH_2_PO_4_, 10 mM EDTA, 0.5 mM K_3_Fe(CN)_6_, 0.5 mM K_4_Fe(CN), 0.1% Triton X-100, 10% methyl alcohol and 0.1% X-Gluc) for 30 min. Plant tissues were incubated at 37 °C for 3–5 h before removing the staining solution. Tissues were steeped in 70% (*v*/*v*) ethanol until the chlorophyll was completely removed, replacing ethanol every 2 h. Images were taken with a phase microscope (Leica DM2500).

### 4.4. Protein Production

*OsFH3* cDNA (3663 bp) was generated using RNA extracted from flower using Trizol reagent (Invitrogen, Carlsbad, CA, USA) and ReverTra Ace-α-First Strand cDNA synthesis kit (TOYOBO, Osaka, Japan). The fragments encoding OsFH3 FH2 (bp 2437–3660 with no polyproline region) and OsFH3 FH1–FH2 (bp 2119 to 3660 with four polyproline regions) were amplified, and all three sequences were confirmed by sequencing primers in Supplemental Table S1. These two fragments were cloned into a modified pFastBac-HTB vector (Invitrogen, USA), which added rTEV protein protease cleavage site and 6×his tag, as well as the maltose-binding protein (MBP) to promote protein stability.

Plasmids were transformed into *Escherichia coli* strain Origami B (DE3) (Novagen, Germany), grown shaking at 37 °C until OD_600_ reached 0.6–0.8 when isopropyl–β–d-thiogalactoside was added to a concentration of 0.4 mM, and cells were incubated at 16 °C overnight to induce protein expression. Cultures were collected by centrifugation and resuspended in binding buffer (400 mM NaCl and 40 mM PBS, pH 8.0), followed by the affinity purification using a Ni-NTA resin based on the manufacturer’s manual (Novagen, Madison, WI). The purified proteins were dialyzed overnight against buffer TK (5 mM Tris, 50 mM KCl, 0.5 mM DTT, and 0.5 mM EDTA) and frozen immediately in liquid nitrogen. Protein concentrations were assayed using the Bradford reagent (Bio-Rad, Hercules, CA, USA) with BSA as standard.

Actin was isolated from rabbit skeletal muscle acetone powder based on the method described by Pardee and Spudich [55] and labeled by pyrene iodoacetamide [56,57] or Oregon-Green 488 iodoacetamide [58] on Cysteine 374 or Biotin-actin [59], as required.

### 4.5. Actin Nucleation Assays

Actin nucleation assays were conducted according to the methods described previously [60]; 2 µM monomeric actin (10% pyrene-labeled) was incubated with different concentrations of OsFH3 FH2 or FH1–FPH2 proteins at room temperature in G buffer (5 mM Tris-HCl, 0.1 mM CaCl_2_, 0.1%NaN_3_, 0.2 mM ATP, 0.5 mM DTT, pH 7.0), and at the end, 10 × KMEI buffer (0.5 mM Tris-HCl, 0.1 mM CaCl_2_, 0.2 mM ATP, and 0.5 mM DTT, pH 7.0) was added. Actin polymerization was initiated immediately, and the reaction was allowed to continue for up to 30 min. Pyrene fluorescence, which indicated actin nucleation, was monitored every 5 min measured by Tecan multimode microplate reader, and wavelength 365 nM was detected.

### 4.6. Time-Lapse Microscopy of AF Elongation

The assays were performed essentially as described previously [27,61]. The flow cells were made as described [62]. Forty µM actin monomers (G-actin, 20% Oregon Green-labelled, 4% biotin labeled) was incubated with or without 50 nM OsFH3 FH2 or OsFH3 FH1–FH2 in 2xTRIF buffer (20 mM imidazole pH7.0, 100 mM KCl, 2 mM EGTA, 2 mM MgCl_2_, 100 mM DTT, 0.4 mM ATP, 100 mM CaCl_2_), injected into the flow cell, then immediately observed under an Observer Z1 microscope (Carl Zeiss, Jena, Germany) equipped with an alphaPlanApo x100/1.46-numerical aperture oil objective. These experiments were repeated in the absence and presence of 200 nM profilin.

### 4.7. Actin Filament Depolymerization Assays

The effect of OsFH3 domains on AF depolymerization was performed as described by [63]; 5 µM actin polymers (50% pyrene-labeled) was pre-incubated with different concentrations of OsFH3 FH2 or OsFH3 FH1–FH2 at room temperature for 5 min. The depolymerization reaction was initiated by adding 25-fold G buffer (0.5 mM Tris-HCl, 0.1 mM CaCl_2_, 0.2 mM ATP, and 0.5 mM DTT, pH 7.0) and allowed to continue for 30 min. The decrease of pyrene fluorescence intensity reflecting AF depolymerization was monitored every 5 min by Tecan multimode microplate reader; wavelength 365 nM was detected.

### 4.8. Co-Sedimentation Assays

High- and low-speed co-sedimentation assays were used to determine the AF binding and bundling activities of OsFH3 FH2 and OsFH3 FH1–FH2. Different concentrations of OsFH3 FH2 or OsFH3 FH1–FH2 were mixed with 5 μM AF at room temperature for 30 min in G buffer (0.5 mM Tris-HCl, 0.1 mM CaCl2, 0.2 mM ATP, and 0.5 mM DTT, pH 7.0), followed by centrifugation at 200,000× *g* for 45 min (high-speed assay) [64] or 13,500× *g* for 30 min (low-speed assay) [65] at 4 °C. Supernatants and pellets were separated by 10% SDS-PAGE gel. The gels were stained with Coomassie Brilliant Blue R250 (Sigma-Aldrich, St. Louis, MO, USA), and the amount of actin and FH3 protein in the pellets and supernatants was quantified using ImageJ.

### 4.9. Microtubules Staining of Roots

After fixing and staining of 5-day rice seedling roots, it was cut into thin strips. Then, the samples were observed in 50% glycerol [66]. Leica TCS SP5 confocal laser microscope was used, and 63 times 1.46 aperture HC PLANs objective lens was selected. Anti-β tubulin was used as the first antibody (1:200 dilution) in microtubule staining experiments (Sigma, St. Louis, MO, USA), FITC Anti-mouse IgG (Jackson, MT, USA) acts as a secondary antibody (1:200 dilution).

### 4.10. Colocalization Assays

The fragment encoding the OsFH3 PTEN (aa 1–337) domain and FH1–FH2 (aa 551–1218) domain were amplified from *OsFH3* cDNA (as described above) using primers in Supplemental Table S1, then cloned into pXY104-MCS vector (from Prof. Hongquan Yang’s Lab of SJTU) in frame with the enhanced GFP (eGFP) marker. Sequences of *OsFH5* full length cDNA are from our lab. The AF marker protein FABD_2_ [67] was amplified and fused in-frame to the red fluorescence protein mScarlet [68]. The microtubule marker protein TUA1 [69] was amplified and fused in-frame to the red fluorescence protein mCherry [68]. These constructs were transformed separately into *Agrobacterium tumefaciens* strain GV3101, cultured as previously described [70].

*Agrobacterium*-mediated co-transformation of different constructs into *Nicotiana benthamiana* was performed as previously described [70]. Infiltrated plants were kept in the dark for 30–48 h, when leaves were harvested, and observed in 50% glycerol with Olympus & IXplore SpinSR confocal laser scanning microscope.

The rice protoplasm preparation method was improved according to Zhang et al. [71]. Wild type 9522 seeds germinated and grew for 2 weeks (dark culture), and 50–60 seedlings with removed seeds were selected to prepare protoplasts. First, the material was cut into 0.5 mm pieces, transferred to 0.6 M mannitol solution, and left it in the dark for 10 min. The mannitol solution was removed, the enzymatic hydrolysate (1.5% Cellulase RS, 0.75% Macerozyme R-10, 0.6 M mannitol, 10 mM MES pH = 5.7, 10 mM CaCl2 and 0.1% BSA) added and vacuumed for 1 h, then shaken at 60–80 RPM and incubated in the dark for 4–5 h. The enzymatic hydrolysate was removed, W5 solution (154 mM NaCl, 125 mM CaCl2, 5 mM KCl, and 2 mM MES = pH 5.7) was added, and the protoplasts were released after shaking at room temperature for 1 h in the dark. The W5 solution was filtered by 40 μm Nylon MESH Millipore and washed 3 times with fresh W5 solution at 1500 RPM for 3 min. Protoplasts were collected and precipitated with an appropriate amount of MMG solution (0.4 M mannitol, 15 mM MgCl_2_, and 4 mM MES pH = 5.7) and resuspended to a concentration of 2 × 10^6^ cells/mL. About 10 µg of plasmid DNA and 200 µL of protoplast were added into the 2 mL EP tube; the 220 µL PEG solution was slowly added and mixed gently at room temperature for 20 min. Then, 1 mL of W5 solution was added to dilute the PEG concentration and terminate the reaction, 200 g, acceleration and deceleration set as 1, centrifugation for 2 min. Supernatant was abandoned, 1 mL W5 solution was added to resuspend the protoplasts; these were transferred to a 12-well plate (in advance moistened with 1 mL 5% BSA solution) and dark culture overnight at 22°. The next day, 200 g, acceleration and deceleration set to 1, centrifugation for 2 min. The supernatant was discarded, and the fluorescence signal was observed by a confocal fluorescence microscope.

## Data Availability

The data presented in this study are available on request from the corresponding author.

## Supplementary Materials

The following are available online at https://www.mdpi.com/article/10.3390/ijms222413250/s1.
