# Supplementary material for: OsFH3 Encodes a Type II Formin Required for Rice Morphogenesis
Source: Int J Mol Sci. 2021 Dec 9;22(24):13250. doi: 10.3390/ijms222413250 (PMC8706662; doi:10.3390/ijms222413250)
Supplement: Supplementary file 1 [file ijms-22-13250-s001.zip › Supplemental material.pdf]

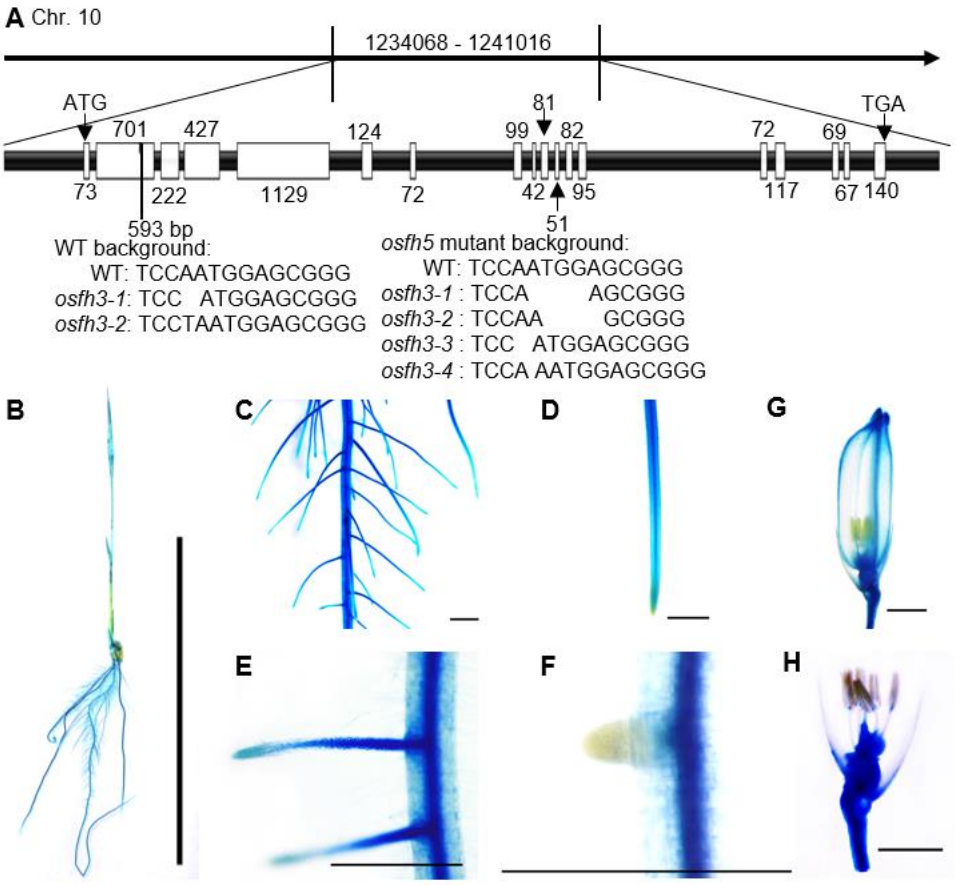

Supplemental Figure S1. *OsFH3* genome structure and *in vivo* expression

(**A**) Structure of genomic *OsFH3*on chromosome 10. 18 exons are indicated with white boxes and the number of bases contained in the exons are indicated numerically, introns and untranslated regions are indicated with the thick black lines. The site and sequences of *osfh3* mutations in exon 2 (593 bp) are shown. (**B**-**H**) Expression of *pOsFH3::GUS* in plant tissues: whole 7-day old seedling (B), roots but not root tips (C-F), and flower but not anthers (G and H). Bars, B = 10 cm; C, D, E, G, H = 1 mm; F = 0.5 mm.

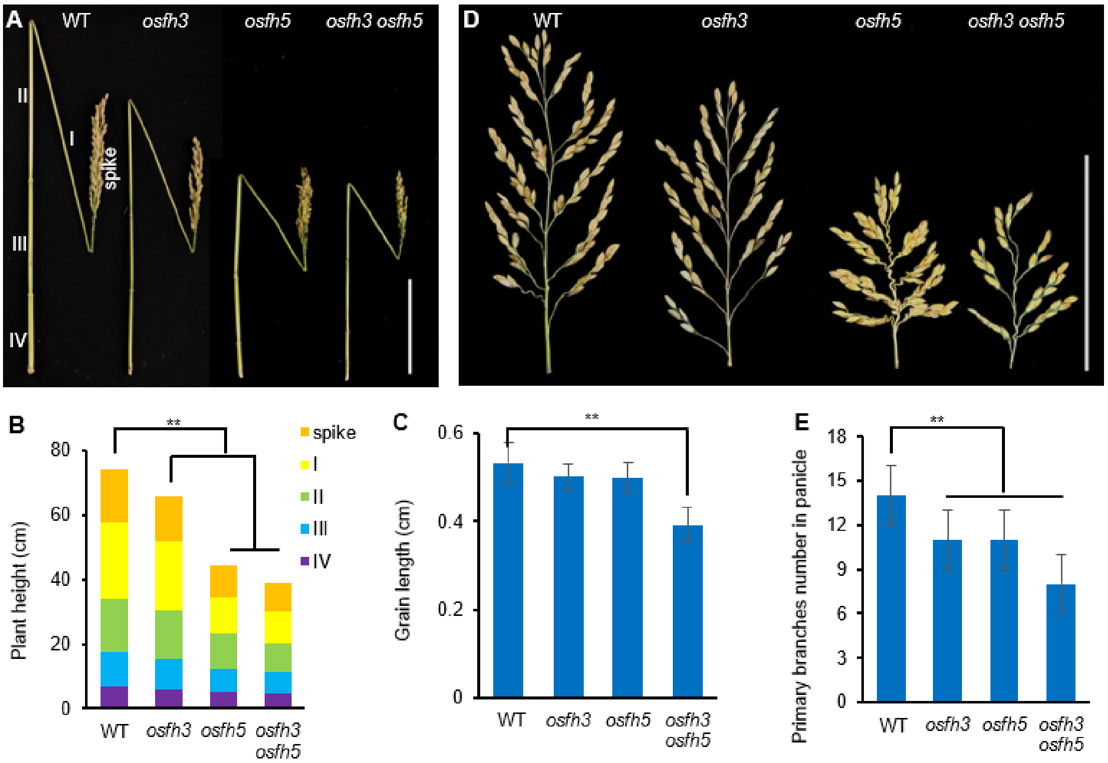

Supplemental Figure S2. Phenotypes of WT, and *osfh3* and *osfh5* single and double mutants

(**A, B**) Rice tiller phenotype (A) and length of internodes I–IV and spike (B, n=20). (**C**) Grain lengths. Mean ± SD, n = 30. ** p<0.01, Student's t-test. (**D**, **E**) Panicle phenotype (D) and primary branch numbers (E). Mean ± SD, n = 18. ** p<0.01, Student's t-test.

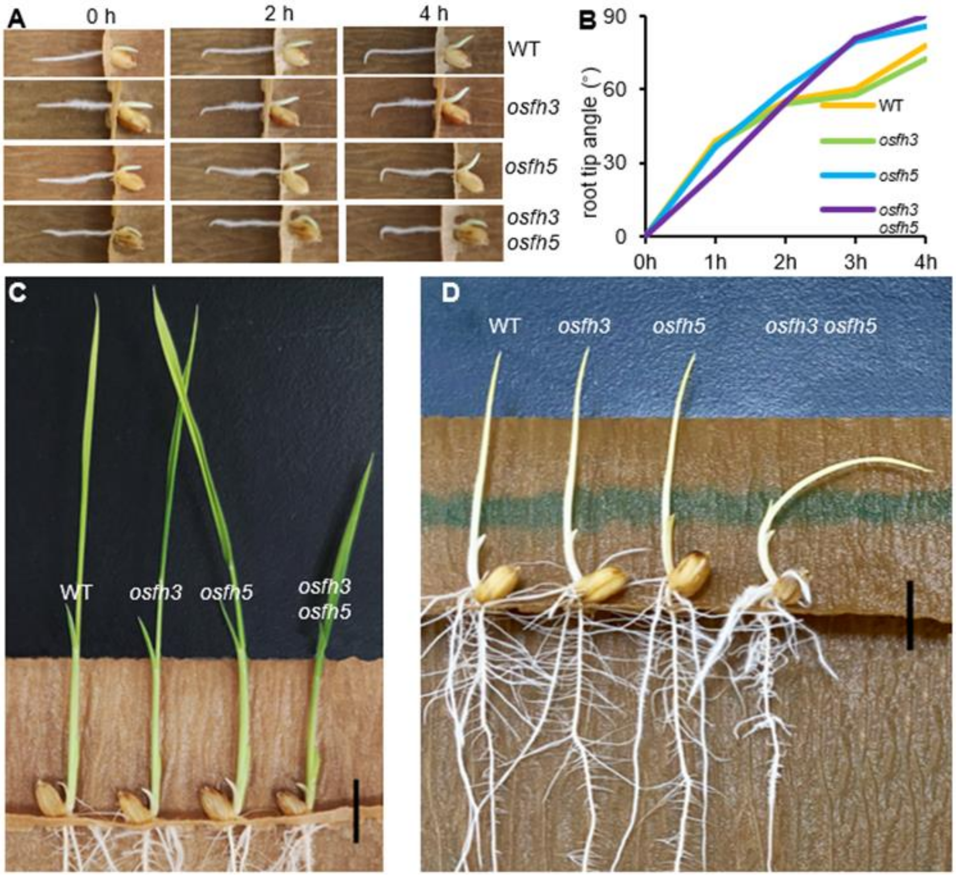

Supplemental Figure S3. osfh3 osfh5 double mutant plants showing altered responses to gravity and light

(**A**) WT*, osfh3, osfh5,* and *osfh3 osfh5* seeds were grown in germination pouches for three days, then rotated 90° to lie horizontally for 4 h. Images shown at 2 h and 4 h after rotation to observe changes in roots tip angles. (**B**) Root tip angles at 1 h increments after rotation to horizontal. N = 16. (**C**, **D**) Growth of WT, *osfh3, osfh5,* and *osfh3 osfh5* plants after 5 days in light (C) and dark (D) conditions. Bars, C = 10cm; D = 1 cm.

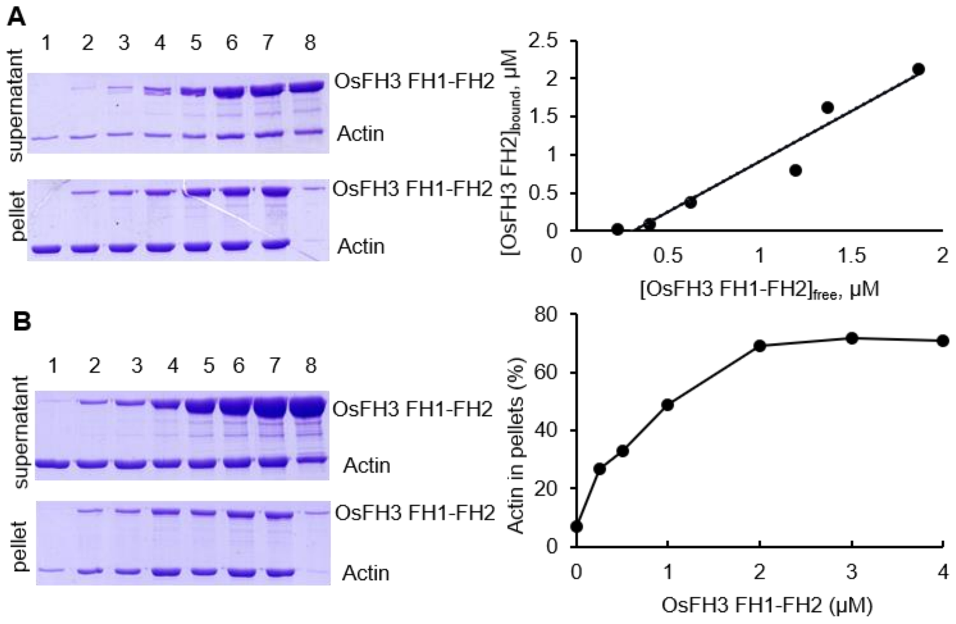

Supplemental Figure S4. OsFH3 FH1-FH2 binds and bundles AF

(**A**) High-speed co-sedimentation assays to determine AF binding to OsFH3 FH1-FH2. Lanes 1–7, 5 μM actin with 0, 0.25, 0.5, 1, 2, 3, 4 μM OsFH3 FH1-FH2, respectively; lane 8, 4 μM OsFH3 FH1-FH2, no actin. Graph (left) shows quantification of Coomassie staining on protein gel (right). (**B**) Low-speed co-sedimentation assays to determine AF bundling with OsFH3 FH1-FH2. Lanes 1–7, 5 μM actin with 0, 0.25, 0.5, 1, 2, 3, 4 μM OsFH3 FH1-FH2, respectively; lane 8, 4 μM OsFH3 FH1-FH2, no actin. Graph (left) shows quantification of Coomassie staining on protein gel (right).

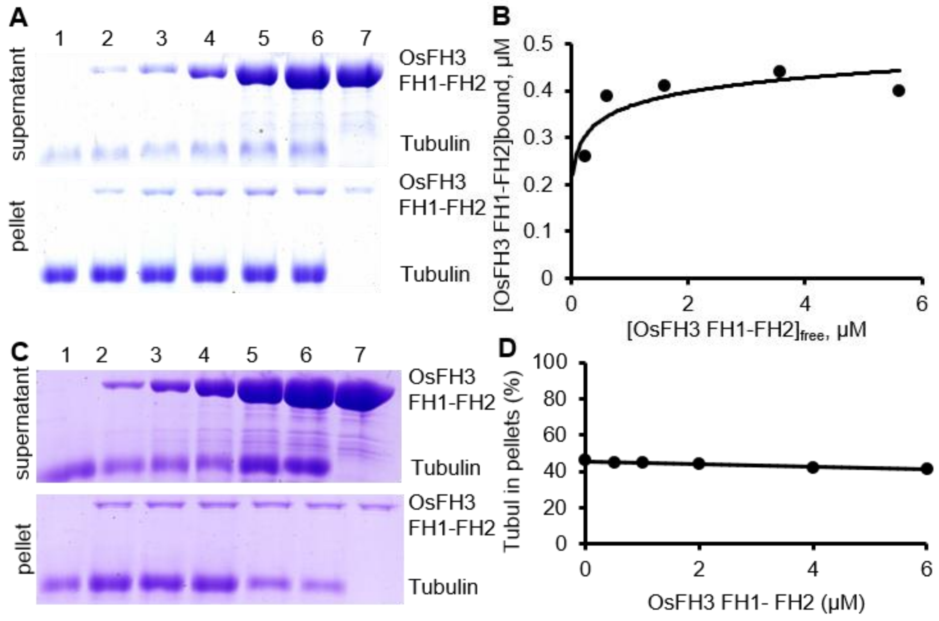

Supplemental Figure S5. OsFH3 FH1-FH2 binds microtubules

(**A**) High-speed co-sedimentation assays to determine tubulin binding to OsFH3 FH1-FH2. Lanes 1–6, 2 µM microtubule with 0.5, 1, 2, 4, 6 µM OsFH3 FH1-FH2, respectively; lane 7, 6 µM OsFH3 FH2, no tubulin. (**B**) Graph shows quantification of Coomassie staining on protein gel (A). (**C**) Low-speed co-sedimentation assays to determine tubulin bundling with OsFH3 FH1-FH2. Lanes 1–6, 2 µM tubulin with 0.5, 1, 2, 4, 6 µM OsFH3 FH2, respectively; lane 7, 6 µM OsFH3 FH1-FH2, no tubulin. (**D**) Graph shows quantification of Coomassie staining on protein gel (C).

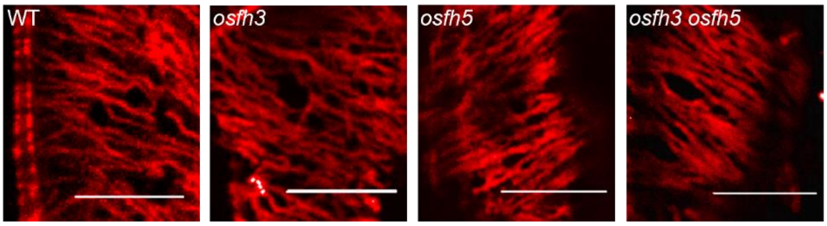

Supplemental Figure S6. Microtubule staining results

Microtubule staining results of WT, *osfh3*, *osfh5* and *osfh3 osfh5* 5days seedlings roots. Bar=10μm.

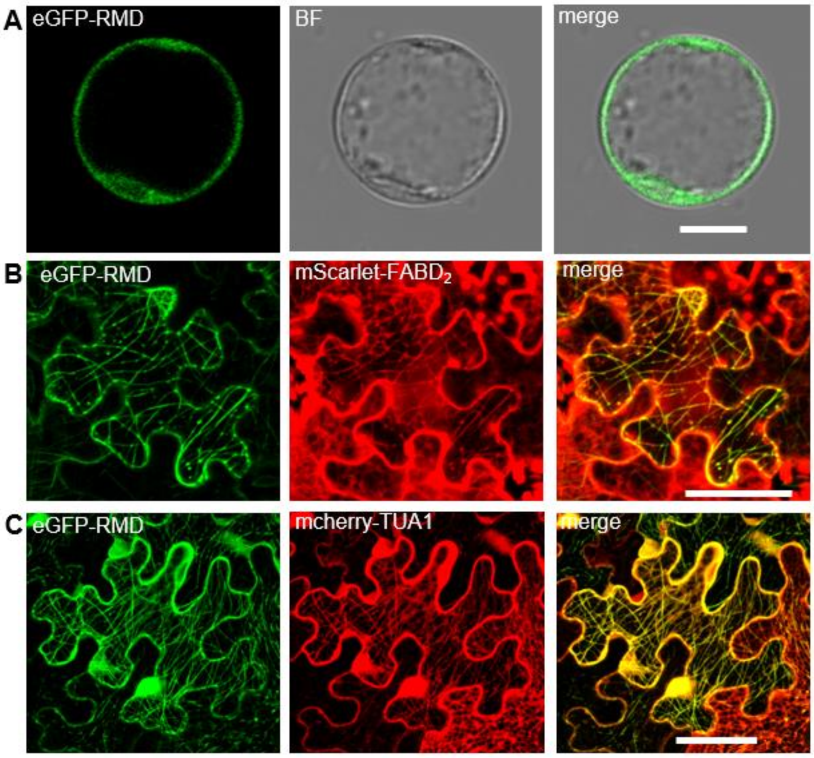

Supplemental Figure S7. OsFH5 colocalizes with microfilaments and microtubules *in vivo*

(**A**) eGFP-OsFH5 expressed in rice protoplast. BF, bright field. Bar=10μm. (**B**) eGFP-OsFH5 and microfilament marker mScarlet-FABD$_2$ co-expressed in tobacco leaves. Bar = 20μm. (**C**) eGFP-OsFH5 and microtubule marker mCherry-TUA1 co-expressed in tobacco leaves. Bar = 20μm.

Supplemental Table S1 Related primers used in this study

OsFH3 cDNA sequencing primers	
Name	Sequence
FH3-1S	GTTTCGATTCCGATAATGGA
FH3-2S	TCGCAGTAATGGCCCTAGT
FH3-2A	TCATCAGGGGAGGAGGTG
FH3-3S	GTGGAGTCCAAACTGAGAGTGT
FH3-4S	GCAAGTGACATCAATTCTGGT
OsFH3 promoter primers constructed into GUS containing vector	
FH3-GUSS	TTACGAATTCGAGCTCGGTACC<br>ACTCCGACTTACCTTTTAGCAATAGT
FH3-GUSA	TTTACCCTCAGATCTACCAT<br>TTCATATGAATGAATAGATCTTCTAAATGT
OsFH3 promoter sequencing primers	
PFH3-1A	CTGATGGGACGCCTATGTG
PFH3-1-1S	TCATGTGCCTTGTGGGGA
PFH3-1S	TAATTCTAATGCCATGTAAGCG
PFH3-2S	AGGGTGAGAGAGAGCGTGAT
OsFH3 recombinant proteins expression primers	
MBPFORMIN3-1S	CTGTATTTTCAGGGCGAATTC<br>GCTAATCGCAGTAATGGCCC
MBPFORMIN3-A	TGATGGTGATGGTGATGAAGCTT<br>TTCCAAGACTTTCTTTGCAGACA
MBPFORMIN3-2S	CTGTATTTTCAGGGCGAATTC<br>CAGCAAAGTAACCCTCCAAAGA
Full length and segments of OsFH3 proteins infused with eGFP primers	
FH3gfpS	AATTACAGGTACCCGGGGATCC<br>ATGTCACTGCTTAGTAGATTCTTCTACAAG

FH3gfpA	CGCCGTCGACTCTAGAGGATCC TTACTTGTACAGCTCGTCCATGC	
FH3-FH1FH2	AATTACAGGTACCCGGGGATCC	ATG
gfpS:	CCATCTGTCCTACCTCCCACG	
FH3-PTEN gfpA	GCCCTTGCTCACCATACTAGT CTCACAAAACAGCATCTCTGCTC	
